# Diagnostic Odyssey: Primary CNS Lymphoma (Lymphoma Cerebri) Presenting as Rapidly Progressive Dementia

**DOI:** 10.1155/crh/8819799

**Published:** 2025-07-09

**Authors:** Yuanli Lei, Aqsa Asharf, Jessica Amos, Mohamad Khawandanah, Anand Annan, James Battiste, Michel Torbey, Taha Al-Juhaishi, Nidhiben Anadani

**Affiliations:** ^1^Department of Medicine, The University of Oklahoma Health Sciences Center, Oklahoma City, Oklahoma, USA; ^2^Section of Hematology-Oncology, Department of Medicine, The University of Oklahoma Health Sciences Center, Oklahoma City, Oklahoma, USA; ^3^Department of Neurology, The University of Oklahoma Health Sciences Center, Oklahoma City, Oklahoma, USA; ^4^Department of Pathology, The University of Oklahoma Health Sciences Center, Oklahoma City, Oklahoma, USA

## Abstract

Primary central nervous system lymphoma (PCNSL) is a rare brain cancer that sometimes presented as rapidly progressive dementia. Diagnosing PCNSL presenting with rapidly progressive neurocognitive symptoms can be challenging, especially when the patient was previously treated with immunosuppressants for suspected autoimmune processes. We present a case where PCNLS was eventually and successfully treated 18 months after neurological symptoms started.

## 1. Introduction

Primary central nervous system lymphoma (PCNSL) is a rare primary brain cancer with an estimated incidence rate of 0.45 per 100,000 population [1]. PCNSL has been increasingly recognized to share clinical features in common with Creutzfeldt–Jakob disease (CJD), the leading cause and an important differential diagnosis of rapidly progressive dementia (RPD) [2]. Lymphomatosis cerebri, a rare variant of PCNSL, has been reported to mimic CJD with atypical clinical presentations and imaging findings [3]. Pathological examination of brain tissue is the gold standard for diagnosing PCNSL and CJD [4, 5]. Nonetheless, diagnosing PCNSL in patients presenting with rapidly progressive neurocognitive symptoms can be challenging as glucocorticoids and other immunosuppressants, often used as a treatment for other differential diagnoses, can alter the natural history of PCNSL, and increase the risk of a nondiagnostic biopsy. We report a case of PCNSL presenting with atypical symptoms overlapping with features of CNS demyelinating disorders, along with an extremely challenging diagnosis process clouded by false-positive 14-3-3 protein and real-time quaking-induced conversion (RT-QuIC).

## 2. Case Presentation

A 69-year-old female with a past medical history of rheumatoid arthritis, breast cancer treated with bilateral mastectomy 10 years before admission and hormonal therapy, and essential tremor was admitted with progressive neurological deterioration. The patient reported progressive cognitive deterioration, increased somnolence (sleeping 14–16 h daily before admission), and short-term memory loss for the last 3 months. She started using a walker due to the new onset of bilateral lower extremity weakness.

Eighteen months before admission, the patient noticed initial symptoms of fatigue, nausea, vomiting, gait instability, and a tendency to fall toward her right.

**Figure 1:**
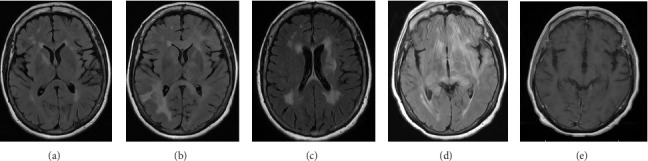
MRI images of different stages during disease course. (a): Imaging at symptoms onset. FLAIR sequence, white matter signals in the right temporal lobe, right anterior limb of the internal capsule/basal ganglia, and the corona radiata concerning for demyelinating lesions or acute disseminated encephalomyelitis. (b): Interim imaging after receiving methylprednisolone. FLAIR sequence, increased size of the right temporal lobe lesion, and multiple new areas of contrast enhancement in the periventricular area. (c): Interim imaging after receiving rituximab. FLAIR sequence, decreased size of the right peritrigonal lesion, an increased size of the lesion in left corona radiata extending into corpus callosum, basal ganglia, and subinsular region along with patchy enhancement and new enhancing lesions in the right thalamus and parietal cortex. (d): Imaging at admission. Postcontrast T1-weighted, worsening of known supratentorial lesions, and extension of lesions in infratentorial areas with new enhanced lesions in bilateral pericallosal/cingulate gyrus region, left corona radiata, left basal ganglia, and right lateral parietal lobe. (e): Imaging after hematopoietic cell transplant. Postcontrast T1-weighted, resolution of previously seen enhancing lesions.

Magnetic resonance imaging (MRI) of the brain with and without contrast (Figure 1(a)) from an outside facility showed white matter signals in the right temporal lobe, right anterior limb of the internal capsule/basal ganglia, and the corona radiata concerning for demyelinating lesions or acute disseminated encephalomyelitis (ADEM). Her cerebrospinal fluid (CSF) analysis ruled out infectious etiologies. Oligoclonal bands were not detected in her CSF. ADEM was the favored diagnosis considering the patient reported a recent infection with influenza. The patient received five doses of intravenous methylprednisolone 1 g daily with improvements in her symptoms at her follow-up visit.

However, repeated brain MRI (Figure 1(b)) 3 months later showed the increased size of the right temporal lobe lesion and multiple new areas of contrast enhancement in the periventricular area, mainly in the left frontal and parietal lobe. This patient experienced worsening imbalance symptoms. She was treated again with 3 days of intravenous methylprednisolone. The patient was referred and evaluated by neuro-oncology and neuro-immunology 9 months before admission with persistent fatigue, new-onset bilateral lower extremity weakness, and short-term memory loss. Her CSF analysis showed increased protein (68 mg/dL, reference range: 17–50 mg/dL) and negative for oligoclonal bands, but increased myelin basic protein (11.8 ng/mL, reference range: 0.0–4.7 ng/mL). Other workups include normal blood complement components 3 and 4, C-reactive protein, rheumatoid factor, angiotensin-converting enzyme, soluble interleukin-2 levels, and a negative serum autoimmune encephalitis/paraneoplastic panel. Serum myelin oligodendrocyte glycoprotein antibody and aquaporin-4 antibody were negative. A repeat brain MRI 3 months later showed two new lesions with contrast enhancement and persistent contrast enhancement in the right temporal lobe lesion. MRI of the spinal cord was normal. Skull base to mid-thigh PET/CT did not show focal abnormal FDG activity. A stereotactic brain biopsy was performed. Pathological examination and immunohistochemical stain showed a focal, macrophage-rich white matter lesion of unspecific etiology, favoring an active inflammatory demyelinating disorder. The patient was treated with a loading dose of rituximab (1 g every 2 weeks for two doses) with a working diagnosis of neuromyelitis optica spectrum disorder with negative aquaporin-4 antibody, with symptom improvements.

Repeat MRI brain (Figure 1(c)) 3 months after rituximab treatment showed a decreased size of the right peritrigonal lesion, an increased size of the lesion in left corona radiata which is now extending into corpus callosum, basal ganglia, and subinsular region along with patchy enhancement in these areas, and new enhancing lesions in the right thalamus and parietal cortex. CSF from repeated lumbar puncture showed protein 60 mg/dL (reference range: 17–50 mg/dL), negative oligoclonal band, and myelin basic protein 9.3 ng/mL (reference range: 0.0–4.7 ng/mL). Three months before admission, the patient reported persistent diplopia. She was treated with cyclophosphamide and intravenous immunoglobulins.

The patient took propranolol chronically for her essential tremor. Her tremor has worsened with her current illness, which prompts propranolol dose escalation.

On admission, the patient appeared frail and chronically ill. Her vital signs were within normal limits. The patient had severe word-finding difficulty/expressive aphasia. She could verbalize minimally with single words. She was wheelchair-bound. During her speech and swallow evaluation, severe oropharyngeal dysphagia and silent aspiration on any liquid consistency were noted in the modified barium swallow study. She received enteral nutrition through a nasogastric tube and later a percutaneous gastrostomy.

On examination, the patient was alert and oriented to self and place only. Bilateral lower extremity strength was normal, but with increased muscle tone, unable to relax with passive movements. Left upper extremity Hoffman sign was positive. The deep tendon reflex was 3+ at the patellar and left ankle. Mild unsustained myoclonus was noted at the right ankle. Cross abductor reflexes were present. Plantar reflexes were equivocal.

Following admission, a CT chest, abdomen, and pelvis with contrast showed no signs of malignancy. MRI brain with and without contrast (Figure 1(d)) showed worsening of known supratentorial lesions and extension of lesions in infratentorial areas with new enhanced lesions in bilateral pericallosal/cingulate gyrus region, left corona radiata, left basal ganglia, and right lateral parietal lobe. There are new areas of enhancement along the ependymal margins of lateral ventricles. Her CSF from repeat lumbar puncture tested at the National Prion Disease Pathology Surveillance Center returned positive for RT-QuiC, and 14-3-3 GAMMA (9154, AU/mL, reference range 173–1999 AU/mL), but negative for Tau protein (580 pg/mL, reference range 0–1149 pg/mL), suggesting 98% likelihood of prion disease. Diagnostic EEG noted prominent, broad, sharp waves over the left frontal region, which occurred in periodic runs at 1 Hz, and rare independent broad, sharp waves over the right frontal region. Findings were suggestive of an increased epileptogenic potential possibly secondary to a structural lesion in the left frontal region, with mild, diffuse encephalopathy.

**Figure 2:**
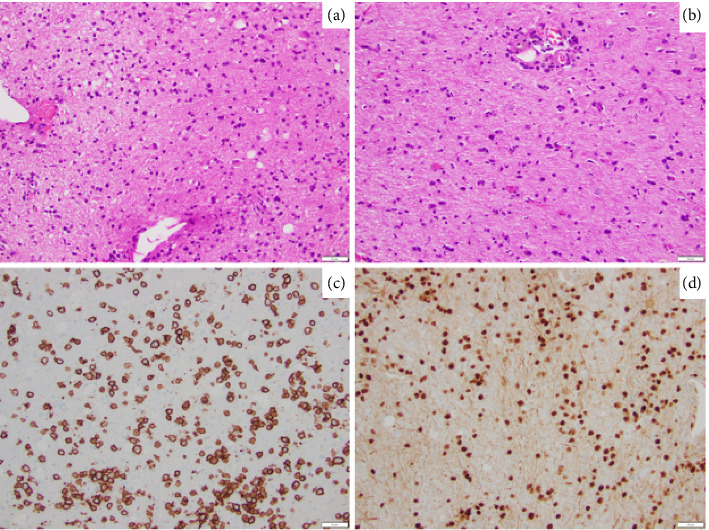
Histopathologic examination and immunohistochemistry stains of brain biopsy specimen. (a): Discohesive infiltrate of atypical cells that are intermediate in size with moderately condensed chromatin. (b): Atypical cells diffusely involving the glial parenchyma without an angio-centric distribution. (c): Immunohistochemical stain of CD20. (d) Immunohistochemical stain of PAX5.

A repeat stereotactic brain biopsy of the left frontal lobe was performed. Histopathologic examination of the biopsy showed a discohesive infiltrate of atypical cells that are intermediate in size with moderately condensed chromatin (Figure 2(a)). Of note, these atypical cells were diffusely involving the glial parenchyma without an angiocentric distribution (Figure 2(b)). Panel of immunohistochemical stains performed shows these cells to be positive for CD20 (Figure 2(c)), PAX5 (Figure 2(d)), BCL6, and MUM1, with increased Ki67 proliferation index, and is negative for CD10, BCL-2, c-MYC, CD5, cyclin D1, and EBV in situ hybridization. The above immunophenotype is compatible with an atypical B-cell infiltrate. To assess and establish clonality, molecular immunoglobulin heavy chain gene rearrangement testing was performed and was positive; in addition, *MYD88 L265P* mutation testing was also positive. The above findings are consistent with involvement by a large B-cell lymphoma. To further classify, FISH testing for *MYC*, *BCL-2*, and *BCL6* rearrangements was performed and was negative. Given the absence of systemic lymphoproliferative disorder, along with the above pathology findings, this was classified as primary diffuse large B-cell lymphoma of the CNS, nongerminal center subtype.

A neurology consultation was obtained. They concluded that the RT-QuIC and 14-3-3 results were likely false positives, given the atypical clinical presentation and imaging findings. The patient was treated with intravenous methotrexate 3000 mg/m^2^ with a total dose of 5310 mg on hospitalization Day 9. A sample from her second brain biopsy was sent to the National Prion Disease Pathology Surveillance Center, where immunostaining with 3F4, the monoclonal antibody to the prion protein returned negative, not supporting a diagnosis of CJD, after discharge. The patient continued receiving methotrexate-based chemotherapy every 14 days. Rituximab was added from Cycle 3 and forward. Temozolomide was added on Cycle 7. During follow-up, the patient had significant improvement in aphasia and dysphagia. She could answer questions with a single sentence during Cycle 2 of chemotherapy (Day 23). After evaluation by a speech-language pathologist, the patient was cleared for solids with thin liquids through the mouth. After Cycle 7 of chemotherapy, the patient was able to walk on a treadmill unassisted for 10 min.

Patient underwent autologous hematopoietic cell transplant rescue with carmustine and thiotepa after 8 cycles of chemotherapy. Brain MRI (Figure 1(e)) at follow-up 2 months after autologous hematopoietic cell transplant showed resolution of previously seen enhancing lesions suggesting no active disease. PET/CT skull base to mid-though showed no FDG-avid lesions. Her PCNSL is considered in complete remission.

## 3. Discussion

PCNSL is a rare, aggressive extra-nodal lymphomatous malignancy affecting the brain, spinal cord, leptomeninges, or vitreoretinal space, without evidence of systemic involvement [6]. Initial presenting symptoms vary widely. About 40%–50% of PCNSL may develop nonspecific cognitive or behavioral changes over weeks to months. It has been increasingly recognized and reported that PCNSL can present as RPD [7–10]. Approximately 50%–70% of PCNSL will present focal neurological deficits [11]. Contrast-enhanced MRI is the most sensitive and informative imaging modality for diagnosing PCNSL [12]. Characteristics MRI findings include isointense-to-mildly hyperintense on T1-weighted imaging, hypointense on T2-weighted images, and almost always with enhancement [13]. Lesions can be unifocal or multifocal, usually periventricular.

Disease course and major events of our case are listed in Table 1. As seen in our case, it is critical to differentiate PCNSL from other causes of RPD, if possible, at an early stage of the disease, as partial treatment with immunosuppressants, most commonly glucocorticoids could alter clinical course, imaging findings, and diagnostic workup yield, making diagnosing PCNSL at a later stage challenging. Meta-analysis suggested patients with RPD due to PCNSL present more commonly with impaired memory, apathy, abnormal speech, and gait, without headache, seizure, or myoclonus, compared with case series of patients with RPD from CJD and other non-PCNSL etiologies as control [14]. However, differentiate RPD etiologies based on symptoms is not sensitive nor specific. Due to its invasive nature, brain tissue biopsy is often, if not always, considered the last resort of diagnostic workup, commonly occurring after empirical treatment failure. Our case is especially challenging as findings of serial brain MRI were atypical and CSF analysis was inconclusive.

RPDs refer to a group of heterogeneous disorders that include immune-mediated, infectious, metabolic encephalopathies, neoplastic as well as prion diseases, and atypically rapid presentations of more common neurodegenerative diseases [2, 3, 15]. The majority of patients with RPD progress from independence to complete (or near-complete) dependence within 1–2 years from the first disease-related symptoms onset [16]. A retrospective study reported that MR imaging findings alone were able to suggest a specific cause of RPD in only 32% of RPD cases [17]. In clinical practice, after exhaustion of noninvasive diagnostic modalities (laboratory and imaging studies), we often resort to invasive tissue sampling (e.g., CSF analysis and brain biopsy) to assist in diagnosing.

Prion diseases are considered prototypical and leading causes of RPD. Based on a longitudinal multicenter study utilizing autopsy or clinical diagnostic criteria, prion diseases were found in 34% of patients with RPD [18]. Prion diseases are a group of rapidly progressive neurodegenerative disorders with long incubation periods. Protein-containing particles (PrP^Sc^) replicate by autocatalytic templating, replacing normal prion proteins (PrP^C^) causing conformational conversion of the normal cellular prion protein into an abnormal misfolded pathological form and leading to neurotoxicity. Currently, prion diseases are universally fatal without effective treatment [19]. Among prion diseases, sporadic CJD (sCJD) is the most common human prion disease, with an incidence of 1-2 cases per 1,000,000 individuals worldwide [20]. It is generally regarded as a spontaneous neurodegenerative illness, arising from either spontaneous prion protein gene (*PRNP*) somatic mutation or stochastic prion protein structural change [2]. Other variants of CJD could be inherited or related to exposure. Cognitive symptoms (memory loss, dysphasia/aphasia, frontal lobe symptoms, confusion, disorientation, etc), cerebellar symptoms (gait, limb ataxia, etc), and behavioral symptoms (agitation/irritation, depression, etc) are most commonly the first symptoms of sCJD. Other common symptoms and clinical findings include constitutional symptoms (asthenia/fatigue, headache, malaise, vertigo/dizziness, altered sleep, and eating patterns), visual disturbances, extrapyramidal features, and myoclonus [21]. On brain MRI, the most sensitive findings of sCJD are hyperintensities within the basal ganglia, the thalamus, and cortex [13]. Clinical diagnosis of sCJD is based on the constellation of symptoms and ancillary tests, including CSF, EEG, and brain MRI. A definitive diagnosis of sCJD requires a neuropathological examination of brain tissue. RT-QuiC testing exploits the intrinsic ability of trace-amount PrP^Sc^ in testing specimens to promote the conformational rearrangement of PrP^C^ substrate that can aggregate into amyloid fibrils. A positive CSF RT-QuIC has been reported with a sensitivity of 71.4%–97.4% and a specificity of 98%–100% among studies [22–25]. Since its original introduction in 2011, a probable diagnosis of sCJD can be made with positive RT-QuIC in CSF in the context of neuropsychiatric disorder based on diagnostic criteria by the United States Centers of Disease Control and Prevention [26]. Despite its intrinsic high sensitivity and specificity, the positive predictive value of a positive CSF RT-QuiC is affected by its testing population. Hermann et al. reported five false-positive cases using CSF RT-QuiC in 371 control patients with nonprion diseases [27]. Four of the five patients who were tested false positive had autoimmune/paraneoplastic encephalitis. Other false-positive CSF RT-QuiC cases include status epilepticus [28], frontotemporal lobar degeneration [24], a pediatric leukemia patient receiving intrathecal chemotherapy [29], and steroids-responsive encephalopathy [30]. It appears that many of the reported false-positive cases occurred in the setting of CNS inflammation, although the exact underlying mechanism is lacking based on current literature.

The 14-3-3 protein, on the other hand, is a normal neuronal protein released into CSF in association with acute neuronal injury [31]. False-positive results have been observed in numerous disorders with rapid neuronal loss, including acute stroke, encephalitis, and other dementing disorders [32]. Therefore, it is not a specific test for evaluating sCJD in an unselected population.

In our case, our patient's initial MRI findings were mostly involving white matter. On the contrary, prion diseases typically cause gray matter. The patient's second brain biopsy pathology contained findings compatible with PCNSL, evidenced by immunohistochemistry, immunoglobulin gene rearrangement, and *MYD88 L265P* mutation corresponding to lymphoid malignancies. She demonstrated significant clinical and imaging improvement following systemic chemotherapy targeting CNS lymphoma. Her initial symptoms started 18 months ago, while the median survival of sCJD from symptom onset is 5 months [33]. For 8 months following her diagnosis of PCNSL, there has been no progression of symptoms related to prion's diseases. Given the low incidence of sCJD and other prion diseases, we feel our patient having concurrent PCNLS and prion diseases is extremely unlikely. Her elevated RT-QuiC and 14-3-3 GAMMA in CSF were likely falsely positive due to neuronal loss secondary to PCNSL. Our case highlights the challenging diagnosis process for PCNSL, especially in patients presented with atypical clinical or imaging changes and treated with prior immunosuppressants. It also emphasizes recognizing that when applying tests even with extremely high specificity in an unselected population, false positive would occur.

## Figures and Tables

**Table 1:** Disease course and major events.

Time	Event
18 months	Symptoms started: fatigue, nausea, vomiting, gait instability, tendency to fall toward right side MRI brain showed findings concerning for ADEM Treated with pulse dose steroids for 5 days with mild symptom improvement

15 months	Patient reported double vision Repeat MRI brain showed new enhancing, periventricular lesions Treated with pulse dose steroids for 3 days with symptom improvement

9 months	Symptoms progressed with nausea, vomiting, fatigability Evaluated by neuro-oncology MRI brain showed new contrast-enhanced lesions Skull to mid-thigh PET/CT did not show focal abnormal FDG activity Brain biopsy favor inflammatory demyelinating disease

8 months	Treated with rituximab, with symptom improvements; oral prednisone taper

5 months	Repeat MRI brain showed improvement of right peritrigonal lesion, worsening of left corona radiata lesion, along with new enhancing lesions

3 months	Reported persistent diplopia; treated with cyclophosphamide and IVIG

Admission	Rapidly progressive cognitive decline, dysphagia, and speech difficulty. CSF tested positive for 14-3-3, RT-QuiC Repeat brain biopsy concerned for lymphoma, with further testing confirming diagnosis of primary diffuse large B-cell lymphoma of the CNS Treated with the first cycle of intravenous methotrexate on Day 9

+ 6 months	Autologous hematopoietic stem cell transplant rescue following carmustine and thiotepa

+ 8 months	PCNSL in complete remission based on brain MRI and PET/CT

## Data Availability

The data that support the findings of this study are available from the corresponding author upon reasonable request.
